# One-year results of a clinical trial of olipudase alfa enzyme replacement therapy in pediatric patients with acid sphingomyelinase deficiency

**DOI:** 10.1038/s41436-021-01156-3

**Published:** 2021-04-19

**Authors:** George A. Diaz, Simon A. Jones, Maurizio Scarpa, Karl Eugen Mengel, Roberto Giugliani, Nathalie Guffon, Isabela Batsu, Patricia A. Fraser, Jing Li, Qi Zhang, Catherine Ortemann-Renon

**Affiliations:** 1 Icahn School of Medicine at Mount Sinai, New York, NY USA; 2 St Mary’s Hospital, Manchester University Foundation Trust, University of Manchester, Manchester, UK; 3 Regional Coordinating Center for Rare Diseases, University Hospital Udine, Udine, Italy; 4 Clinical Science for LSD, SpinCS, Hochheim, Germany; 5 Dept Genetics, UFRGS, Medical Genetics Clinical Research Group, HCPA, and INAGEMP, Porto Alegre, Brazil; 6 Reference Center for Inherited Metabolic Disorders, Femme Mère Enfant Hospital, Lyon, France; 7 Sanofi, Bridgewater, NJ USA; 8 Sanofi Genzyme, Inc, Cambridge, MA USA

## Abstract

**Purpose:**

To assess olipudase alfa enzyme replacement therapy for non–central nervous system manifestations of acid sphingomyelinase deficiency (ASMD) in children.

**Methods:**

This phase 1/2, international, multicenter, open-label trial (ASCEND-Peds/NCT02292654) administered intravenous olipudase alfa every 2 weeks with intrapatient dose escalation to 3 mg/kg. Primary outcome was safety through week 64. Secondary outcomes included pharmacokinetics, spleen and liver volumes, lung diffusing capacity (DL_CO_), lipid profiles, and height through week 52.

**Results:**

Twenty patients were enrolled: four adolescents (12–17 years), nine children (6–11 years), and seven infants/early child (1–5 years). Most adverse events were mild or moderate, including infusion-associated reactions (primarily urticaria, pyrexia, and/or vomiting) in 11 patients. Three patients had serious treatment-related events: one with transient asymptomatic alanine aminotransferase increases, another with urticaria and rash (antidrug antibody positive [ADA+]), and a third with an anaphylactic reaction (ADA+) who underwent desensitization and reached the 3 mg/kg maintenance dose. Mean splenomegaly and hepatomegaly improved by >40% (*p* < 0.0001). Mean % predicted DL_CO_ improved by 32.9% (*p* = 0.0053) in patients able to perform the test. Lipid profiles and elevated liver transaminase levels normalized. Mean height *Z*-scores improved by 0.56 (*p* < 0.0001).

**Conclusion:**

In this study in children with chronic ASMD, olipudase alfa was generally well-tolerated with significant, comprehensive improvements in disease pathology across a range of clinically relevant endpoints.

## INTRODUCTION

Acid sphingomyelinase deficiency (ASMD) (historically Niemann–Pick disease [NPD] types A [OMIM257200], B [OMIM607616], A/B) results from pathogenic sequence variants in the *SMPD1* (EC3.1.4.12) gene encoding the lysosomal enzyme acid sphingomyelinase (ASM). While ASM metabolic pathways and consequences of deficient enzyme are complex,^1^ sphingomyelin is the primary storage compound. Intracellular sphingomyelin accumulation is especially damaging in lipid-laden macrophages of the reticuloendothelial system, and in the most severe cases in neurons of the central nervous system.^2^ Systemic manifestations of ASMD include hepatosplenomegaly, bleeding/bruising, thrombocytopenia, dyslipidemia, interstitial lung disease (ILD), delayed growth and puberty, osteoporosis/osteopenia, liver dysfunction with progressive fibrosis, and cardiac disease.^3,4^

ASMD prevalence is 0.4–0.6/100,000 births with chronic forms more common.^5^ Subtypes reflect a disease spectrum ranging from uniformly fatal ASMD type A (infantile neurovisceral disease)^6^ to chronic forms ASMD type B (chronic visceral) and ASMD type A/B (chronic neurovisceral). ASMD type B is associated with little or no progressive neurological involvement, while ASMD type A/B has neurologic characteristics much less severe than those observed in ASMD type A.^3,7^ Chronic ASMD frequently begins in childhood and is associated with significant morbidity and sometimes early mortality due to respiratory or liver failure.^8^ Pediatric patients with chronic forms of ASMD may be at particular risk for early death.^9,10^

ASMD management relies on supportive care in the absence of an approved, disease-modifying therapy.^11^ Enzyme replacement therapy (ERT) with olipudase alfa (recombinant human ASM, which does not cross the blood–brain barrier) is in clinical development to treat non–central nervous system manifestations of ASMD. Olipudase alfa was well tolerated in phase 1 trials in adults with chronic ASMD after implementing a within-patient dose escalation regimen.^12,13^

This paper describes safety and tolerability (primary objective), pharmacokinetics (PK), pharmacodynamics (PD), and exploratory efficacy results of a clinical trial of olipudase alfa in pediatric patients with chronic ASMD ranging in age from infancy to adolescence.

## MATERIALS AND METHODS

### Study design/participants

This phase 1/2, open-label, single-arm study (NCT02292654/EudraCT2014–003198–40) was conducted at six sites (Brazil, France, Germany, Italy, United Kingdom, and United States) from 1 May 2015 to 9 December 2019. Site institutional review boards approved the protocol and patients/parents provided written consent. Pediatric patients with confirmed ASMD, spleen volume ≥5 multiples of normal (MN), and height *Z*-score ≤ −1 were eligible and recruited sequentially in three cohorts beginning with adolescents (12 to <18 years), then children (6 to <12 years), and infants/early children (<6 years).

Exclusion criteria included mean platelet counts of <60 × 10^9^/L, alanine aminotransferase (ALT) or aspartate aminotransferase (AST) > 250 IU/L (same in U/L) total bilirubin >25.7 µmol/L (>1.5 mg/dL), or an international normalized ratio >1.5. Individuals with acute or rapidly progressive neurological abnormalities and/or with the following genotypes associated with ASMD type A were excluded: homozygous or heterozygous combinations of the *SMPD1* variants c.1493G>T (p.Arg498Leu), c.911T>C (p.Leu304Pro), or c.996delC (p.Phe333SerfsTer52) (*SMPD1* reference sequence NM00543.4, Gen-BankNG_011780.1).

### Olipudase alfa infusions

Olipudase alfa infusions were administered once every 2 weeks starting at 0.03 mg/kg followed by 0.1 mg/kg, two consecutive doses of 0.3 mg/kg, two consecutive doses at 0.6 mg/kg, single doses of 1 mg/kg, 2 mg/kg, and 3 mg/kg (maintenance dose). Patients were monitored as inpatients for at least 24 hours postinfusion during dose escalation. Dosing schedules were adjusted per safety and protocol prespecified dose-limiting toxicity criteria (defined in Supplemental Table A). Premedication was administered per investigator discretion to manage infusion-associated reactions (IARs).

### Outcomes

Safety and tolerability through week 64 were assessed by monitoring of treatment-emergent adverse events (including IARs), physical and neurological examinations, vital signs, electrocardiograms, clinical laboratory tests including liver function, safety biomarkers (including ceramide, high-sensitivity C-reactive protein, ferritin, calcitonin, interleukin 6 [IL-6] and interleukin 8 [IL-8]). Immunogenicity was assessed using an enzyme-linked immunosorbent assay (ELISA) for IgG antidrug antibody (ADA) (minimum titer: 50; 1:50 dilution). Positive samples were evaluated for neutralizing antibodies.

Plasma olipudase alfa concentrations were determined using ELISA (lower limit of quantitation 39 ng/mL) and PK parameters calculated following the first 0.3, 1, and 3 mg/kg doses and at week 52 using noncompartmental methods. Plasma sphingomyelin and metabolites were quantitated by liquid chromatography–tandem mass spectrometry. Chitotriosidase activity was assayed using fluorogenic substrate molecules and normalized based on *CHIT1* genotype (patients homozygous for the common null variant resulting in no enzyme activity were excluded from analysis and values for heterozygotes were doubled).

Exploratory efficacy outcomes through week 52 included spleen and liver volume assessed by magnetic resonance imaging (MRI), lung disease scoring using high-resolution computed tomography (HRCT) scans, and chest X-ray.^14^ Pulmonary function testing^15^ including lung diffusing capacity for carbon monoxide (DL_CO_)^16,17^ was conducted only in patients ≥5 years of age able to perform tests using acceptability criteria (e.g., inspired volume during DL_CO_ testing had to be at least 85% of the vital capacity recorded from spirometry testing). Plasma lipid profiles, platelet counts, and height *Z*-scores were also assessed.

### Analyses

Analyses were performed overall and by age group. Descriptive statistics were used for continuous variables and for concentration time data. Categorical variables were summarized using frequency and percent. Change from baseline and percent change from baseline were analyzed using a regression model with baseline as covariate. Least square means and 95% confidence intervals were determined. No multiplicity adjustments were conducted. All *p* values are nominal.

Abdominal MRI and HRCT pulmonary images were read by external central providers blinded to patient and time point. Pulmonary imaging of lung regions was qualitatively assessed based on a 4-point scale (0 = no interstitial disease; 1 = mild [1–25% lung volume affected]; 2 = moderate [26–50%]; 3 = severe [51–100%]). DL_CO_ was expressed as percent predicted values corrected for hemoglobin (>80% was considered normal/no impairment, >60% to ≤80% mild impairment, 40% to 60% moderate impairment, and <40% severe impairment).^18^ Liver and spleen absolute volumes were calculated as multiples of normal (MN) assuming normal spleen and liver volumes of 0.2% and 2.5% of body weight, respectively.^19^ Severe and moderate organomegaly were defined as >15 and >5 to ≤15 MN, respectively, for spleen and >2.5 and >1.25 to ≤2.5 MN, respectively, for liver.^19^

## RESULTS

### Patient baseline characteristics

**Fig. 1:**
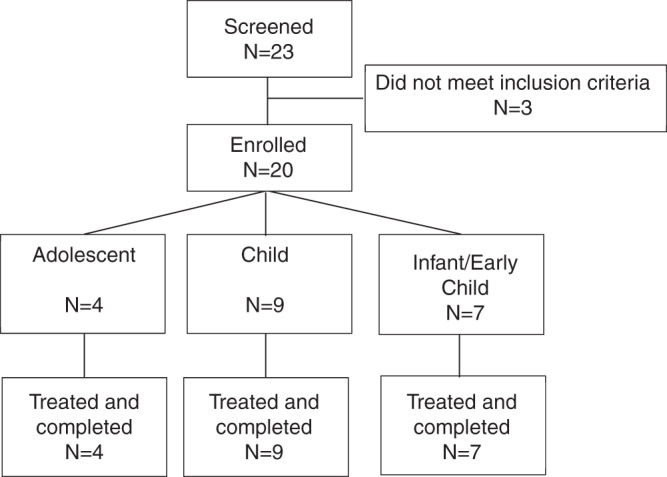
Patient Disposition in the Trial. Patient disposition.

Twenty patients were enrolled and completed the trial (Fig. 1). Baseline characteristics are shown in Table 1. Age ranged from 1.5 to 17.5 years, males and females were equally represented, and the majority were Caucasian. Median age at symptom onset and diagnosis were 1.0 and 1.9 years, respectively. At disease onset, most patients had organomegaly and 10–35% also presented with respiratory disease, excessive bleeding/bruising, and/or thrombocytopenia. At baseline, 12 patients (60%) had severe splenomegaly (>15 MN). The study did not differentiate between patients with ASMD type B or A/B. No enrolled patient had characteristics of ASMD type A.

**Table 1:** Demographics and baseline characteristics by age group and overall population.

	Adolescent	Child	Infant/early child	Overall
	(*N* = 4)	(*N* = 9)	(*N* = 7)	(*N* = 20)
	Number of patients (%)			
Age (years)				
Mean (SD)	14.84 (2.22)	8.69 (1.69)	3.77 (1.44)	8.20 (4.39)
Median	14.8	8.72	3.75	7.99
Min:Max	12.32:17.46	6.00:11.52	1.49:5.57	1.49:17.46
Sex, *n* %				
Female	3 (75)	4 (44)	3 (43)	10 (50)
Male	1 (25)	5 (56)	4 (57)	10 (50)
Race, *n* (%)				
White	3 (75)	7 (78)	7 (100)	17 (85)
Southeast Asian	1 (25)	1 (11)	0	2 (10)
Other	0	1 (11)	0	1 (5)
Ethnicity, *n* (%)				
Hispanic/Latino	0	0	1 (14)	1 (5)
Not Hispanic/Latino	4 (100)	9 (100)	6 (86)	19 (95)
Age at symptom onset (years)				
Mean (SD)	1.370 (0.581)	1.633 (1.295)	1.170 (0.903)	1.420 (1.017)
Median	1.277	1.187	0.985	1.027
Min:Max	0.84:2.09	0.38:3.92	0.16:2.54	0.16:3.92
Age at diagnosis (years)				
Mean (SD)	2.124 (0.708)	3.358 (3.360)	1.551 (1.200)	2.478 (2.449)
Median	1.993	2.839	1.183	1.932
Min:Max	1.42:3.09	0.02:11.09	0.21:3.10	0.02:11.09
ASM activity (peripheral leukocytes) nmol/h/mg				
Mean (SD)	0.210 (0.092)	0.129 (0.061)	0.095 (0.067)	0.135 (0.078)
Median	0.225	0.130	0.085	0.120
Min:Max	0.09:0.30	0.00:0.20	0.03:0.22	0.00:0.30
*SMPD1* variant				
Homozygous for p.Arg610del	0	0	0	0
Heterozygous for p.Arg610del	1 (25.0%)	3 (33.3%)	2 (28.6%)	6 (30.0%)
Other variants	3 (75.0%)	6 (66.7%)	5 (71.4%)	14 (70.0%)
Fully intact spleens	4 (100%)	9 (100%)	7 (100%)	20 (100%)
Severe splenomegaly (>15 multiples of normal)	1 (25.0%)	5 (55.6%)	6 (85.7%)	12 (60.0%)
Severely reduced DLco (<40%)	1 (33.3%)	0	0	1 (11.1%)^a^

^a^Based on nine patients able to perform the test at baseline.

### Dose escalation

All patients reached the maintenance dose of 3 mg/kg and completed the 64-week study. In some, doses were repeated or decreased due to IARs or dose-limiting toxicity (Supplemental Table A). One patient in the infant/early child group temporarily discontinued treatment from weeks 14 to 26 after the 0.6 mg/kg dose infusion due to an anaphylactic reaction as described in detail in the Supplemental Material. This same patient and two other patients in the child group also had infusion interruptions due to urticaria. Seven patients in the infant/early child or child groups had at least one dose reduction/repeat dose prior to reaching the maximum study target dose.

### Safety

Supplemental Table A summarizes the treatment-emergent adverse event profile reported by age group and the overall population and lists events occurring in two or more patients. All patients experienced at least one event, 88% (705/798) of which were mild. Events common to all age groups included pyrexia, cough, vomiting, nasopharyngitis, diarrhea, headache, nausea, rhinitis, oropharyngeal pain, ear pain, and rhinorrhea.

Seventeen percent (136/798) of treatment-emergent adverse events were considered related to treatment, 75% (102/136) of which were identified as IARs (the majority of which were urticaria, pyrexia, and vomiting) (Supplemental Table A). No IARs were reported in the adolescent group. Overall, 88% of IARs were mild (90/102) and 98% (100/102) occurred within 72 hours of infusion (33 during infusion, 6 from 0–3 hours postinfusion, 29 between 3–24 hours postinfusion, and 32 from 24–72 hours postinfusion). One severe IAR event was associated with an anaphylactic reaction (see below and Supplementary Material).

Five patients (25%, one in child group and four in infant/early child group) experienced at least one serious adverse event (12 total), all of which resolved. Five serious events in three patients in the infant/early childhood group were considered treatment-related/possibly related. One event was a serious anaphylactic reaction in a 17-month-old patient that occurred during infusion at the 0.6 mg/kg dose. The patient temporarily discontinued treatment and subsequently tested positive for IgG and IgE ADA. Dose escalation was resumed after desensitization and the target dose was reached. A narrative is provided in Supplementary Material. This patient also had a serious event of gastroenteritis considered unrelated to treatment. The four additional serious events considered drug-related were two transient asymptomatic ALT increases in one patient (who also had a serious event of increased ALT considered unrelated), and one case each of urticaria and rash in another patient. The remaining five unrelated serious events were gastroenteritis in one patient in the infant/early childhood group and events of mycoplasma pneumonia, respiratory failure, talipes, and fracture in one patient in the child group.

There were no clinically significant abnormalities in laboratory findings, vital signs, electrocardiograms, or echocardiograms. Ten events met criteria for dose-limiting toxicity (defined in Supplemental Table A) in six patients, primarily during dose escalation, and all resolved after temporary dose reductions or repeat dosing.

Twelve patients were positive for IgG ADA post-treatment (including one positive at baseline with a titer of 50). Another patient was positive for ADA at baseline but had no treatment-induced changes. Among eight patients with a persistent ADA response (negative for ADA at baseline) all had a low response (titers ≤ 400) except the 17-month-old patient with the anaphylactic reaction (peak titer: 1,600; intermediate response). Among ADA-positive patients, none tested positive for neutralizing antibody that interfered with enzyme uptake into cells and one tested transiently positive for inhibition of enzyme catalytic activity. ADA status had no impact on olipudase alfa PK as shown by the similar mean total drug exposure (AUC_0-t_) at baseline and week 52 for patients positive and negative for treatment emergent ADA responses (Supplementary Figure A).

At baseline, transaminase levels were abnormal in 16/20 (80.0%) patients. Mean baseline levels of AST and ALT (± SD) in the adolescent, child, and infant/early child groups (56.3 ± 27.3, 80.6 ± 46.4, and 104.4 ± 66.0 IU/L, and 52.0 ± 16.4, 62.9 ± 38.1, 69.4 ± 33.0 IU/L, respectively; values the same in U/L) were elevated relative to normal ranges, consistent with hepatic involvement in ASMD. While there were postinfusion transient increases in some liver function tests defined as dose-limiting toxicities resulting in temporary dose reductions, changes were asymptomatic and not clinically significant. Liver transaminase levels normalized during treatment (see below).

### Safety biomarkers

**Fig. 2:**
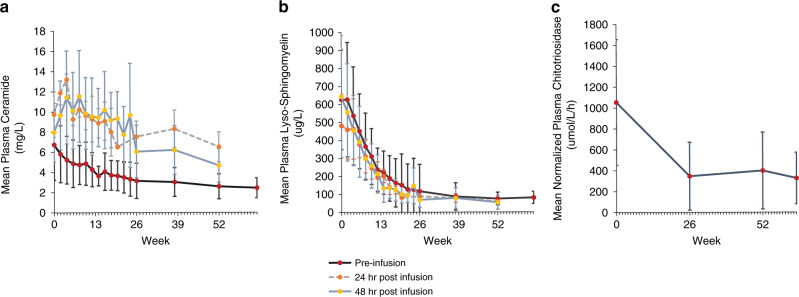
Safety Biomarker and Pharmacodynamic Parameters. Plasma levels of ceramide, lyso-sphingomyelin, and chitotriosidase activity during treatment with olipudase alfa. (**a**) Mean (±SD) plasma levels of ceramide for the overall patient population preinfusion, 24 hours and 48 hours after olipudase alfa infusions. Normal range for plasma ceramide was 1.3–3.3 mg/L. 24 hour postinfusion levels were assessed in the adolescent group only.  (**b**) Mean (±SD) plasma levels of lyso-sphingomyelin for the overall patient population preinfusion, 24 hours and 48 hours after olipudase alfa infusions. Upper limit of normal for lyso-sphingomyelin is 9.99 μg/L. Twenty-four hour postinfusion levels were assessed in the adolescent group only. (**c**) Mean (±SD) normalized plasma activity of chitotriosidase for the overall patient population. Upper limit of normal is 181 μmol/L/hr.

Plasma ceramide (normal range: 1.3–3.3 mg/L) was used to monitor rapid debulking of sphingomyelin during dose escalation. Mean plasma ceramide level (±SD) at baseline was 6.7 ± 3.5 mg/L overall and varied slightly across age groups (7.1 ± 2.1, 5.6 ± 1.9, and 8.0 ± 5.3 mg/L in the adolescent, child, and infant/early child groups, respectively). Mean ceramide levels increased transiently following all olipudase alfa infusions compared with preinfusion levels (Fig. 2a). Both pre- and postinfusion ceramide levels declined at each dosing step and after patients had attained the maintenance dose, suggesting that the debulking strategy of escalating doses helped manage ceramide release.

There were no safety signals for calcitonin. Transient increases in levels of C-reactive protein, ferritin (and decrease in iron), IL-6, and/or IL-8 with pyrexia and vomiting during dose escalation in three patients were characterized by investigators as acute phase reactions and led to dose adjustments at the next infusion.

### Pharmacokinetics

PK parameters [C_max_, t_max_, AUC_last_, AUC(_0-τ_), CL, V_ss_, and t_1/2z_] were generally similar across age groups. Olipudase alfa exposures after 3 mg/kg dosing were similar after the first dose and at week 52 within each age group, indicating minimal accumulation with repeat infusions every 2 weeks. Summary descriptive statistics of PK parameter values for the 3 mg/kg dose are provided in Supplementary Table B.

### Pharmacodynamics

Lyso-sphingomyelin (a deacylated form of sphingomyelin) and chitotriosidase (secreted by activated macrophages) are both elevated in plasma of patients with ASMD (ULN 9.99 µg/mL and 181μmol/L/hr, respectively).^13,20^ These biomarkers were monitored to assess olipudase alfa pharmacodynamic effects. Mean baseline plasma lyso-sphingomyelin level (±SD) was 626 ± 277 µg/L overall and was similar in the child and infant/early child groups (654 ± 226 and 670 ± 382 µg/L, respectively), and lower in the adolescent group (488 ± 153 µg/L). Both pre- and postinfusion levels were similar and stable decreases from baseline were noted in all patients in the first weeks of olipudase alfa treatment, with the majority of the overall decrease occurring during the first 26 weeks (Fig. 2b).

Chitotriosidase activity was elevated at baseline in the overall population and the mean ± SD percent decrease from baseline to week 52 in the overall group was −58.0 ± 24.8% (Fig. 2c). Mean ± SD percent decrease from baseline to week 52 in each age group was −55.8 ± 21.1% in the adolescent group, −44.7 ± 25.0% in the child group, and −74.6 ± 18.1% in the infant/early child group.

### Exploratory efficacy

#### Spleen and liver volume

**Fig. 3:**
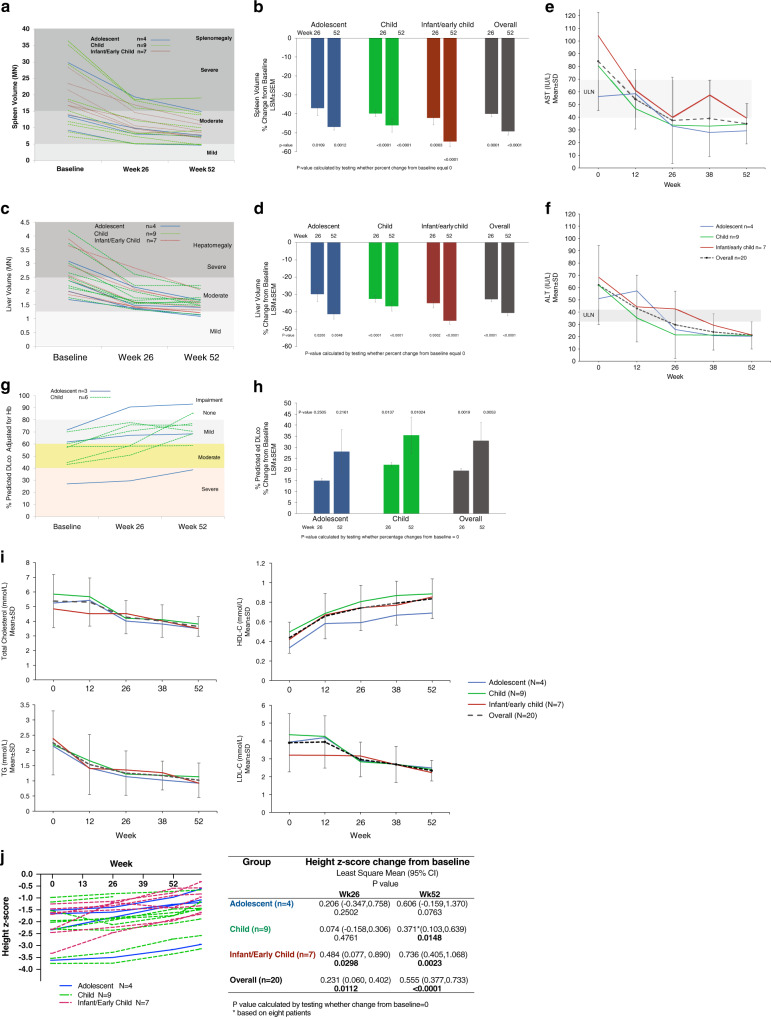
Changes over time in efficacy assessments (hepatosplenomegaly, liver transaminases, lung diffusion capacity, plasma lipids, and height) with olipudase alfa treatment. (**a**, **b**) Individual patient responses for spleen volumes in multiples of normal (MN) and the percent change from baseline for spleen volumes in least square mean (LSM) ± standard error of the mean (SEM), respectively at 6 months and 1 year. Cutoffs of MN for severity categories are indicated by shading. (**c**,**d**) Individual patient responses for liver volumes in MN and the percent change from baseline for spleen and liver volumes in LSM ± SEM, respectively at 6 months and 1 year. Cutoffs of MN for severity categories are indicated by shading. (**e**,**f**) Mean (±SD) preinfusion plasma levels for aspartate aminotransferase (AST) and alanine aminotransferase (ALT), respectively, for the overall patient population and the means for each age group at baseline and throughout treatment with olipudase alfa. The AST and ALT upper limit of normal (ULN) for the for the adolescent, child, and infant/early child groups is 40, 59, and 69 IU/L and 43, 34, and 34 IU/L, respectively (values the same in U/L). Ranges of ULN are indicated by gray boxes. (**g**, **h**) individual patient responses and LSM ± SEM percent change from baseline for the percent predicted DL_CO_ adjusted for hemoglobin. Cutoffs for gas exchange impairment are indicated. (**i**) Plasma lipid mean (±SD) for the overall population and means for each age group for total cholesterol, low density lipid cholesterol (LDL-C), high density lipid cholesterol (HDL-C), and triglycerides (TG). Low to high ranges of normal for total cholesterol: 4.4–5.15 mmol/L ( <170–199 mg/dL). Low to high ranges of normal for LDL-C: adolescent: 1.60 to 3.52 mmol/L, child: 1.63 to 3.63 mmol/L, infant/early child: 0.98 to 3.63 mmol/L (38–140 mg/dL); for TG: adolescent: 0.36 to 1.67 mmol/L, child: 0.34 to 1.48 mmol/L, infant/early child: 0.34 to 1.24 mmol/L (32–149 mg/dL); for HDL-C: adolescent: 0.78 to 1.92 mmol/L, child: 0.93 to 1.94 mmol/L, infant/early child: not reported (30–75 mg/dL). (**j**) Individual patient height *Z*-scores (graph) and the LSM changes from baseline with *p* values (inset table) over time.

All patients had moderate or severe splenomegaly and hepatomegaly at baseline, with improvement seen at the first assessment at week 26 (Fig. 3a, c).

Individual decreases in spleen volume ranged from −23% to −61%, and 11/12 patients with severe splenomegaly at baseline improved to moderate levels by week 52, including six patients in the infant/early child group (Fig. 3a). Mean spleen volume in the overall population decreased by −49.2 ± 9.7% (*p* < 0.0001) from 19 ± 8.8 MN at baseline to 9.3 ± 3.9 MN at week 52. Decreases in spleen volumes over time were similar in the three age groups, with mean percent changes from baseline of −46.9%, −46.0%, and −54.6% in the adolescent, child, and infant/early child groups at week 52, respectively (Fig. 3b). The mean percent changes from baseline were statistically significant in all age groups at both week 26 and week 52 (Fig. 3b).

All ten patients with severe hepatomegaly at baseline improved to moderate levels by week 52 (Fig. 3c). Individual decreases in liver volume ranged from −17% to −61%. Mean liver volume decreased by −40.6 ± 9.4% at week 52 (baseline: 2.2 ± 0.7 MN, week 52: 1.5 ± 0.3 MN). Mean decreases over time were similar across age groups and mean percent changes from baseline were statistically significant at both week 26 and week 52 (Fig. 3d).

Evidence for portal hypertension was assessed in 18 patients by liver ultrasound Doppler. Normal direction of blood flow through the portal vein was preserved in all patients, consistent with the absence of portal hypertension.

#### Liver function tests

Preinfusion levels of liver enzymes and bilirubin were monitored over time. With treatment, elevated mean baseline levels of transaminases normalized in all age groups (Fig. 3e, f). In the overall population, the mean percent change from baseline (±SD) at week 52 was −51.9 ± 15.2% for AST and −59.4 ± 21.9% for ALT. At baseline, AST and ALT were abnormal in 80% (16/20) of patients while at week 52, 2/19 patients had abnormal AST and 2/20 had abnormal ALT levels.

Mean total bilirubin levels were within normal ranges at baseline and throughout the study (data not shown). One patient had an abnormal value at baseline and no patient had an abnormal value at week 52.

#### Lung Anatomy and function

Baseline mean ± SD ILD scores confirmed moderate ILD: 2.3 ± 1.0, 2.6 ± 0.6, and 2.5 ± 0.8 in the adolescent, child, and infant/early child groups, respectively. At week 52, reductions were observed in ground glass appearance and/or reticulonodular density, and mean ILD scores ± SD decreased to 2.0 ± 0.5, 2.0 ± 1.1, and 1.9 ± 1.3 in the adolescent, child, and infant/early child groups, respectively. Moreover, severe ILD observed at baseline in six patients decreased to mild or moderate in five and was absent in one by week 52. Chest X-ray results also showed measurable improvements from baseline (data not shown).

The 13 patients at least 5 years of age able to perform pulmonary function tests had below normal mean ± SD % predicted FVC, FEV_1_, and TLC tests at baseline of 77.5 ± 16.2%, 76.5 ± 16.1%, and 86.8 ± 23.3%, respectively. Results of tests improved at week 52 to 85.7 ± 17.3%, 81.7 ± 14.0%, and 110.2 ± 24.0%, respectively.

Nine patients were able to complete DL_CO_ testing at baseline (Fig. 3g). Baseline DL_CO_ was severely impaired in one patient, moderately impaired in four, and mildly impaired in four. Individual percent changes from baseline ranged from 0.7% to 91.7%. DL_CO_ improved from baseline in all but two patients in the child group, one with mild and one with borderline mild severity. The overall mean ± SD % predicted DL_CO_ at baseline was 54.8 ± 14.2% and 71.7 ± 14.8% at 1 year, a mean increase of 33% from baseline (*p* = 0.0053) (Fig. 3h).

#### Plasma lipid levels

Baseline lipid profiles varied by age group, but in general mean total cholesterol, low-density lipoprotein, and triglyceride levels were above normal limits, and high-density lipoprotein levels were below normal limits (Fig. 3i). Olipudase alfa treatment was associated with improvements in lipid levels by week 26. By week 52, mean lipid levels were within normal limits. Apolipoprotein A1 and B levels also improved, and lipoprotein(a) levels did not change over time (data not shown).

#### Platelet counts

Mean ± SD baseline platelet counts were 137.74 ± 62.32, 98.93 ± 9.28, 148.82 ± 87.50, and 145.66 ± 28.01 × 10^9^/L and increased at week 52 to 173.61 ± 60.46, 143.63 ± 46.63, 172.94 ± 65.42, and 194.58 ± 61.43 × 10^9^/L for the overall, adolescent, child, and infant/early child groups, respectively (normal value 150 × 10^9^/L). The percent change from baseline at week 52 was statistically significant in the overall population (LSM ± SEM 34.03 ± 7.63%, *p* = 0.0003), child group (30.67 ± 12.01%, *p* = 0.0378), and infant/early child group (31.76 ± 8.09%, *p* = 0.0172). In the adolescent group, the percent change from baseline was 45.01 ± 26.26% (*p* = 0.2286).

#### Growth

Figure 3j summarizes height *Z*-scores by age group. By week 52, height *Z*-scores improved in 15 patients (79%) and remained the same in 4 (21%). Individual changes ranged from −0.21 to +1.43. Least square mean changes from baseline and *p* values are shown in the table inset to Fig. 3j. Height *Z*-scores ranged from −3.8 to −1.0 at baseline (median − 2.0) and from −3.4 to −0.6 (median − 1.5) at week 52 (mean improvement: 0.56; *p* < 0.0001).

## DISCUSSION

There remains an unmet need for a disease-modifying treatment for chronic forms of ASMD. In children and young adults with chronic ASMD, significant organomegaly is often the most pronounced symptom^21^ and most patients also have progressive pulmonary disease.^14,22^ Thrombocytopenia^3^ and dyslipidemia^4^ are also reported, and children and adolescents often have growth deficits.^23^ There is an increased mortality burden in pediatric patients^9,10^ where liver and lung disease are primary contributors to mortality.^24^

Olipudase alfa was well tolerated in pediatric patients ranging in age from infants to adolescents and there were no permanent discontinuations or study withdrawals due to adverse events. A large proportion of the treatment-emergent adverse events observed during the study reflected symptoms typically reported by ASMD patients, including upper respiratory tract infections and gastrointestinal disorders.^3,10,21,25^ The majority of treatment-related events were mild to moderate IARs, which occurred only in the infant/early child and child groups. These events were managed in some cases with temporary infusion interruptions or dose reductions. IARs including pyrexia, headache, urticaria, and vomiting are not uncommon during or shortly after ERT infusions.^26^

Acute phase reactions associated with transient increases in C-reactive protein were observed in three patients during dose escalation, and managed with temporary dose repetition or reduction. These results are consistent with observations in adult patients with ASMD.^12,13^ It is hypothesized that ceramide, a promotor of cytokine release and inflammation,^27,28^ may mediate this response. In the first-in-human phase 1 study of olipudase alfa in adults with ASMD type B, acute phase inflammatory reactions that were either preceded or accompanied by large increases in plasma ceramide levels occurred 12 to 24 hours after single doses ≥0.3 mg/kg.^29^ The dose-escalation protocol was established following observations in a mouse model of ASMD where initial treatment with several low-dose infusions of rhASM prevented high-dose toxicity.^30^ The within-patient dose escalation regimen, designed to gradually debulk stores of sphingomyelin and mitigate large increases in plasma ceramide that could trigger acute phase reactions, was shown to be as effective in the present study as it was in the phase 1b study in adults with ASMD type B.^12^ Monitoring postinfusion increases in liver function tests was part of prespecified dose-limiting toxicity criteria as debulking of sphingomyelin and corresponding transient increases of ceramide could trigger transient changes in liver function tests. Transient increases in liver function tests in some patients met criteria for dose-limiting toxicities and resulted in temporary dose repetition or reduction, but changes were asymptomatic and not clinically significant.

While anaphylaxis and other severe hypersensitivity reactions were infrequent, access to appropriate medical support to manage such reactions is an important consideration when using olipudase alfa in patients with ASMD.  Furthermore, in one patient with a previous anaphylactic reaction to olipudase alfa, a desensitization regimen was successfully used to allow continuation of therapy. This patient ultimately reached the target maintenance olipudase alfa dose and remains enrolled in the long-term extension trial. All IARs resolved without sequelae and did not prevent dosing at the maintenance olipudase alfa dose. In addition, ADA titers remained low in the pediatric population, and there was no neutralizing antibody production. Two patients had ADA at baseline, which may indicate the presence of cross-reactivity or autoantibodies that have been reported with biotherapeutics, including ERTs.^31^

The PK and PD data observed in the pediatric population are consistent with results reported in adult patients, although PK exposure is lower in pediatric patients compared wit adults.^12^ Olipudase alfa exposure in both pediatric and adult patients increased in an approximately dose-proportional manner with minimal accumulation following repeated olipudase alfa infusions every other week. Decrease in plasma lyso-sphingomyelin levels over time provides evidence that olipudase alfa is engaging its intended target to reduce lysosomal sphingomyelin in biologically relevant tissues.

One year of olipudase alfa treatment resulted in significant improvements in key disease features known to persist or worsen over time in pediatric patients with chronic ASMD including organomegaly,^10,25^ lung function/interstitial lung disease, and dyslipidemia.^4^ Four children with moderately impaired lung diffusing capacity improved to mild or no impairment by week 52, and one adolescent patient with severe impairment improved to borderline moderate. Among nine patients who performed the test at baseline, 5 (55.6%) had a percent predicted DL_CO_ change from baseline ≥15% at week 52. This improvement is consistent with DL_CO_ changes considered clinically meaningful according to ILD and pulmonary fibrosis guidelines.^32–34^ Improvement in lung diffusion capacity was supported by the improvements in ILD scores on HRCT with decreases in both lungs in mean ground glass appearance, ILD, and reticulonodular density. All but one patient with severe splenomegaly had moderate splenomegaly by week 52. Of the 20 patients in this study, 19 (95.0%) had a reduction from baseline in spleen volume (MN) of ≥30.0% at week 52, which is consistent with the therapeutic goals in other lysosomal storage diseases (LSDs) such as 30–50% reduction in spleen volume after 1 year of treatment in patients with Gaucher disease.^19^ Reduction of splenomegaly was accompanied by increased platelet counts in all pediatric groups, reflecting a correction of secondary hypersplenism. Hepatomegaly improved, and patients with severe hepatomegaly improved to moderate levels. Liver transaminases are often elevated in children and adults with ASMD reflecting liver pathology,^25^ and transaminase levels normalized beginning in the first few weeks of olipudase alfa treatment. All patients had growth retardation at baseline per inclusion criteria, and there was a statistically significant mean improvement in *Z*-score at 1 year in the overall population. Changes by age group were not always significant and the small sample size of each age group needs to be considered.

While the study is limited by the absence of a placebo group, in general, patients with the most severe disease at baseline showed the greatest improvements in disease parameters over time. Pediatric patients treated with olipudase alfa showed comprehensive improvements in disease pathology across a range of clinically relevant disease endpoints. Importantly, responses occurred early; clinically significant improvements observed by 6 months were maintained or improved further after 1 year of treatment.

### Conclusions

Olipudase alfa was well-tolerated and associated with clinically meaningful improvements in disease features in pediatric patients aged 1.5 to 17.5 years with chronic ASMD.

## Supplementary information

Supp_TableA

Supp_TableB_Fig A_narrative

## Data Availability

Information regarding data and materials will be made available individually upon request.
