# Supplementary material for: One-year results of a clinical trial of olipudase alfa enzyme replacement therapy in pediatric patients with acid sphingomyelinase deficiency
Source: Genet Med. 2021 Apr 19;23(8):1543–50. doi: 10.1038/s41436-021-01156-3 (PMC8354848; doi:10.1038/s41436-021-01156-3)
Supplement: Supplementary file 2 — Supp_TableB_Fig A_narrative [file 41436_2021_1156_MOESM2_ESM.pdf]

Supplementary Table B. Pharmacokinetic Parameters for Olipudase Alfa in Pediatric Patients Following 3 mg/kg First Infusion and after Week 52 Infusion

**Age Group**	**Olipudase alfa** 3 mg/kg	**n**	C_{max} µg/mL	AUC_{last} µg.h/mL	$AUC_{(0-\tau)}$ µg.h/mL	**CL** mL/h/kg	$t_{1/2z}$ h	V_{ss} mL/kg	t_{max} h	t_{last} h
			Mean±SD (Geometric Mean) [CV%]						median (min, max)	
Adolescent	First Dose	4	28.0 ± 4.88 (27.7) [17]	452 ± 49.7 (450) [11]	478 ± 55.4 (475) [12]	6.34 ± 0.741 (6.31) [12]	17.1 ± 1.15 (17.1) [7]	133 ± 13.5 (132) [10]	3.94 (3.87, 4.08)	74.73 (72.67, 76.13)
	Week 52	4	22.4 ± 1.02 (22.3) [5]	461 ± 28.1 (461) [6]	489 ± 32.7 (488) [7]	6.16 ± 0.411 (6.15) [7]	24.3 ± 2.88 (24.2) [12]	172 ± 11.6 (171) [7]	3.81 (3.67,4.03)	97.03 (97.00, 98.00)
Child	First Dose	9	23.0 ± 3.93 (22.7) [17]	441 ± 97.8 (431) [22]	465 ± 102 (455) [22]	6.75 ± 1.57 (6.59) [23]	23.1 ± 2.11 (23.0) [9]	166 ± 39.1 (162) [24]	4.00 (3.83,7.07)	95.83 (92.73, 98.25)
	Week 52	9	24.4 ± 7.51 (23.5) [31]	482 ± 101 (472) [21]	508 ± 108 (497) [21]	6.16 ± 1.38 (6.03) [22]	23.3 ± 1.42 (23.2) [6]	153 ± 32.7 (149) [21]	4.25 (3.75,9.78)	96.58 (92.55, 99.75)
Infant/ Early Child	First Dose	7	22.1 ± 7.19 (21.2) [33]	412 ± 87.4 (405) [21]	432 ± 93.0 (424) [22]	7.20 ± 1.40 (7.07) [19]	22.6 ± 1.25 (22.6) [6]	165 ± 31.0 (162) [19]	4.13 (3.75,8.0)	96.33 (92.25, 97.40)
	Week 52	7	22.4 ± 4.18 (22.0) [19]	429 ± 62.6 (425) [15]	451 ± 68.2 (446) [15]	6.79 ± 1.08 (6.72) [16]	23.6 ± 1.35 (23.6) [6]	161 ± 20.9 (160) [13]	4.42 (4.08,5.87)	97.58 (90.88, 99.83)

$AUC_{(0-\tau)}$ area under the curve versus time during a dosage interval
AUC_{last} area under the curve versus time from time 0 to t_{last}
CL total body clearance
C_{max} maximum plasma concentration
t_{max} time to reach C_{max}
t_{last} time corresponding to the last observed concentration above the lower limit of quantitation
$t_{1/2z}$ terminal half life
V_{ss} volume of distribution at steady state

Supplementary Figure A. Mean total drug exposure (AUC $_{0\text{-}t}$) at baseline and Week 52 for patients positive and negative for treatment-emergent (TE) anti-drug antibody (ADA) responses

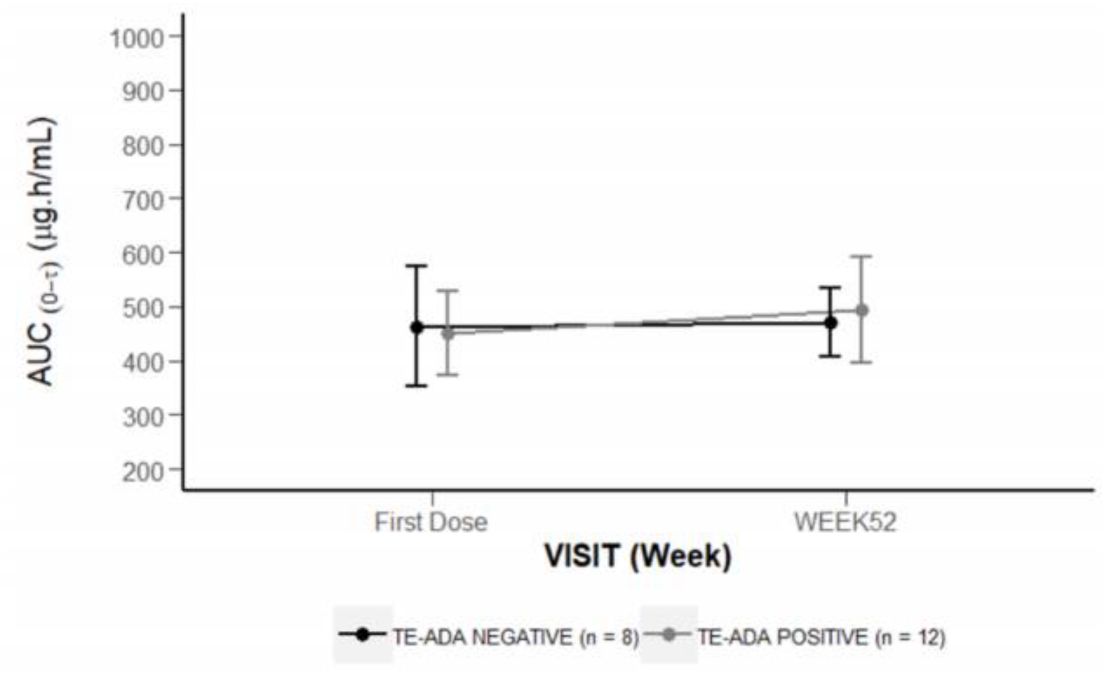

Note: TE as treatment-emergent

Supplementary Information for Diaz et al. "First Clinical Trial of Olipudase Alfa Enzyme Replacement Therapy in Pediatric Patients with Acid Sphingomyelinase Deficiency: One-year Results"

Clinical Description and Management of Patient with Anaphylactic Reaction

Summary

A 17-month-old patient included in the Infant/early child cohort experienced a serious adverse event of urticaria considered an anaphylactic reaction that necessitated stopping treatment until a desensitization protocol was implemented. The patient was heterozygous for two variants previously identified as likely pathogenic, neither of which was the common p.Arg610del variant. Clinical history included allergy or potential allergic reactions for eczema. After undergoing the desensitization protocol, the patient was able to reach the target dose of 3 mg/kg and continues receiving olipudase alfa infusions in the long-term extension study. Intermittent events of vomiting, pyrexia and urticaria have occurred at subsequent post-desensitization infusions, and the patient receives prophylactic treatment with oral diphenhydramine hydrochloride, IV diphenhydramine hydrochloride, and/or IV methylprednisolone at each olipudase alfa infusion. The patient had an intermediate IgG anti-drug antibody (ADA) response and was positive for IgE ADA but did not develop neutralizing antibodies that inhibited either enzyme activity or cellular uptake.

Clinical Details

The patient had events of mild vomiting considered infusion associated reactions (IARs) at the Week 4 visit after receiving 0.3 mg/kg olipudase infusion, at the Week 8 visit after receiving 0.6 mg/kg, and at the Week 10 visit after receiving the second 0.6 mg/kg dose. Vomiting was managed with ondansetron administration. Mild events of pyrexia also occurred post-infusion after the Week 8 and Week 10 visits (body temperature of 38.3°C and 38°C, respectively) and were treated with oral acetaminophen and/or ibuprofen. At the Week 10 visit, the patient also

had an adverse event of urticaria considered an IAR (diffused on face, torso, and extremities) of mild intensity 42 minutes after the start of infusion, during infusion at a rate of 10 mL/hr. Olipudase alfa was temporarily interrupted, and intravenous (IV) diphenhydramine hydrochloride 11 mg (1 mg/kg) administered. The urticaria resolved within 68 minutes, and the olipudase alfa infusion was restarted and completed at a decreased rate of 5 mL/hr. Pre infusion vital signs showed heart rate at 129 beats per minute (bpm), blood pressure (BP) at 116/74 mmHg, and body temperature at 37.0°C. At 1-hour post infusion, heart rate was 121 bpm, BP as 132/86, and body temperature was 38.0°C. Following the event of urticaria, the Investigator decided to repeat the dose of 0.6 mg/kg at the next infusion at Week 12.

At the Week 12 visit, during the infusion of 0.6 mg/kg olipudase alfa, the patient experienced an anaphylactic reaction of severe intensity 27 minutes after beginning infusion during the infusion rate of 10 mL/hr. Heart rate and blood pressure at the time of the event were 186 bpm and 131/71, respectively. The infusion was stopped and corrective treatment administered including intramuscular (IM) epinephrine 0.11 mg, IV methylprednisolone sodium succinate 10.8 mg, and IV diphenhydramine hydrochloride 11 mg. This serious adverse event was assessed as related to olipudase alfa by the Investigator and considered an IAR.

Immunological Assessment

Anti-olipudase alfa seroconversion was observed for this patient at Week 10 (IgG ADA titer=400) and the peak IgG ADA titer during the study was 1600 at Week 12 (corresponding to an intermediate ADA response). At the end of the study (Week 64), the IgG ADA titer was 100. The patient did not develop during the study neutralizing antibodies that inhibited either enzyme activity or cellular uptake.

Additional immunological testing, performed at the Week 12 visit because of the event of anaphylactic reaction of severe intensity, showed positive anti olipudase alfa IgE antibodies pre-infusion (at 1.30 IU/mL; normal reference range <0.35 IU/mL), and post-infusion serum tryptase of 6.7 µg/L (normal reference range ≤12.5 µg/L). Circulating immune complex was 0.8 µg eq/mL post-infusion and complement activation was negative.

Desensitization Protocol and Outcomes

The patient did not receive any olipudase alfa infusions from Week 14 through Week 26 included to allow for preparation and implementation of a desensitization procedure for restarting olipudase alfa infusions.

Olipudase alfa was restarted at a dose of 0.3 mg/kg at Week 28 at a 1:100,000 dilution of the concentration at the time of the anaphylactic reaction, with the addition of a coating solution to prevent adherence of olipudase alfa to the tubing at dilute concentrations. After the first restarting dose, escalation of concentrations proceeded slowly and gradually and was adjusted based on patient tolerance to infusions, which was carefully monitored. As a precautionary measure, Week 28 and Week 30 infusions were done at the pediatric intensive care unit. The patient reached the target maintenance dose of 3 mg/kg at Week 50 and the standard infusion administration procedure was progressively reinstated and fully followed at Week 58.

The patient continued to have IARs of pyrexia and/or vomiting that were mild in intensity at study visits from Week 30 through Week 64. An adverse event of urticaria (scattered) occurred at the Week 32 visit and resulted in stopping the infusion and administration of IM epinephrine 0.12 mg (1 mg/kg), IV diphenhydramine hydrochloride 12.5 mg, and IV methylprednisolone sodium succinate 12.4 mg. Starting from the Week 34 visit, the study

participant received oral diphenhydramine hydrochloride, IV diphenhydramine hydrochloride, and/or IV methylprednisolone as prophylactic treatments at each olipudase alfa infusion.

During the Week 46 visit infusion of a 2.0 mg/kg dose, the patient had an adverse event of urticaria of mild intensity (scattered in the right diaper area, on torso, underarms, calves, and shins) four hours after the start of infusion. Infusion was temporarily interrupted and IV diphenhydramine hydrochloride 13 mg (1mg/kg) was administered. The event of urticaria resolved and the infusion was restarted and completed at the same rate of 20 mL/hr.

Conclusion

Patients showing signs of severe hypersensitivity reactions following olipudase alfa infusions can be successfully managed with a desensitization protocol. Although not routinely recommended, in this case the investigator elected to use pre-treatment prophylaxis with corticosteroids and H1-receptor antagonists to minimize IARs.
